# Differential release kinetics of cardiac biomarkers in patients undergoing off pump coronary artery bypass surgery

**DOI:** 10.1186/1749-8090-8-S1-P156

**Published:** 2013-09-11

**Authors:** A Kapoor, S Singh, SK Agarwal, S Pandey, A Sinha, H Rai, S Kumar, N Garg, S Tewari, PK Goel

**Affiliations:** 1 Cardiology, Sanjay Gandhi PGIMS, Lucknow, India; 2 Cardio-Thoracic Surgery, Sanjay Gandhi PGIMS, Lucknow, India

## Background

B-type natriuretic peptide (BNP) is a routinely used biomarker for heart failure. Levels of BNP increase in patients undergoing open heart surgery. Off pump CABG (OPCABG) has reduced peri-operative morbidity and mortality.

## Methods

We prospectively studied release kinetics of BNP, Troponin-I (TnI) and CKMB 24 hours prior, and 6,24,48 hours and 1 month after OPCABG in 80 patients. (mean age 59.1 yrs, LVEF 52.4%,83% males).

## Results

Baseline BNP, TnI and CKMB levels were 105.8 pg/ml, 0.9 ng/ml and 2.34 ng/ml. Although all biomarkers increased significantly within 6 hours of surgery, release kinetics were different. While peak BNP levels occurred in 96% patients by 24-48 hours, for TnI and CKMB, this was observed in only two-thirds. A trend towards fall in all biomarkers was seen by 48 hours; while TnI and CKMB levels normalized in all at 1 month, 42% patients still had BNP > 100 pg/ml. Those with baseline BNP > 100 pg/ml had lower LVEF (43.6vs55.6%,p<0.01),longer inotrope duration (43.8vs31.4 hrs, p=0.03) and ventilator support time (33.9vs25.6 hrs, p=0.04); mean ICU and hospital stay were similar to those with lower BNP. Despite baseline BNP differences, levels of TnI and CKMB at all times were similar in these two groups. BNP levels had positive correlation with patient age and angiographic Syntax score (p=0.02) and negative correlation with LVEF. Of all biomarkers, only baseline BNP predicted inotrope duration (OR4.9, 95%CI:1.3-17.7,p=.01) and ventilation time (OR4.5,95%CI:1.2-16.6,p=.02). Post-operative levels of BNP at 6,24 and 48 hours and delta BNP were significant predictors of mean ventilation time only.

## Conclusions

Even in patients undergoing OPCABG, there is significant natriuretic peptide (BNP) and myocardial enzyme release (TnI, CKMB) notable within 6 hours of surgery. Patients with higher baseline BNP levels had longer inotrope and ventilator duration. Of all the biomarkers, only BNP had an association with post-operative variables.

